# Exploring the Relationship Between Play During School Recess and Motor Performance in 6- to 8-Year-Old Children

**DOI:** 10.3390/children11111288

**Published:** 2024-10-25

**Authors:** Dagmar F. A. A. Derikx, Marina M. Schoemaker, Leila Faber, Suzanne Houwen, Esther Hartman

**Affiliations:** 1 Department for Human Movement Sciences, University Medical Center Groningen, University of Groningen, Antonius Deusinglaan 1, 9713 AV Groningen, The Netherlands; m.m.schoemaker@umcg.nl (M.M.S.); l.faber02@umcg.nl (L.F.); e.hartman@umcg.nl (E.H.); 2 Inclusive and Special Needs Education Unit, Faculty of Behavioural and Social Sciences, University of Groningen, Grote Kruisstraat 2/1, 9712 TS Groningen, The Netherlands; s.houwen@rug.nl

**Keywords:** motor performance, play, peers, child development, observational

## Abstract

Background: Playing with others, which in school occurs mainly during recess, might be an enabling factor to improve children’s motor performance, as behaviour is shaped by observing and imitating others. Objective: The aim of the current study was to examine whether children’s play activities and with whom 6- to 8-year-old children play during recess are related to their motor performance. Methods: The study sample consisted of 86 Dutch participants (mean age 6.92 ± 0.62 years, 58.1% boys). A modified version of the System for Observing Children’s Activity and Relationships during Play was used to observe the following play variables: sedentary or active behaviour, group size, activity type, and type of interaction. The Movement Assessment Battery for Children 2 was used to measure three components of motor performance: Manual Dexterity, Aiming and Catching, and Balance. Associations between the play variables and the motor components were investigated using compositional data analysis and logistic regressions. Results: The results showed that children who spent more time in sedentary behaviour rather than in active behaviour were less likely to have motor difficulties in the Aiming and Catching component. None of the other play variables were related to motor performance. Conclusions: One explanation might be that these play variables in itself are not related to motor performance, but that these variables should be investigated as an integrated whole rather than in isolation. Therefore, future research should investigate whether interactions between the play variables are related to motor performance.

## 1. Introduction

Motor skills are an important part of children’s daily life, as they are used for a large part of the day [1]. Motor performance can be defined as the execution of learned sequences of movements resulting in a coordinated and efficient action aimed at achieving an intended goal [2,3]. It has been found that 7- and 8-year-old children use their fine motor skills half of the time and their gross motor skills a quarter of the time during a school day [1]. It is, therefore, understandable that motor performance during childhood has been found to be predictive for several factors, such as academic achievement [4] and a physically active lifestyle across one’s lifespan [5,6]. In addition, it has been suggested that motor performance enhances the development of the perceptual, cognitive, and social domains [7]. However, despite its important role, motor performance levels among children have declined significantly over the past few decades [8,9]. Hence, it is crucial to identify modifiable enabling factors that allow for the creation of facilitative situations in which motor performance can be practised and improved.

Play might be an important enabling factor, as it offers children the opportunity to explore their environment and to acquire and further practise their fine and gross motor skills, all while having fun [10,11,12]. Play can be described as a seemingly purposeless activity that is primarily performed for enjoyment and that is unrestricted by the bounds of reality [13]. It can be divided into structured and unstructured play. Structured play involves organized play activities often facilitated by an adult, such as physical education or organized sports. Unstructured play, on the other hand, is spontaneous and child-directed like playing on a playground. Both types of play have been found to improve fundamental motor skills in children [14,15]. As motor performance occurs within a social environment, interaction with others, such as peers, might be another enabling factor [7,16]. According to the social learning theory, behaviour is shaped by observing and imitating the behaviour of others, which is called observational learning [17]. This also applies to motor performance, since children learn new motor skills during interactions with others by first observing how others perform the skill and then practising it themselves [18]. During the large amount of time that children spend in school (on average 376 min/day in the Netherlands [19]), recess is one of the few times that children are allowed to play on the playground and interact with peers relatively freely [20], thus making it a potentially important time to practise and improve their motor skills.

Despite the suggested importance of recess for practising motor skills, to our knowledge, only a few studies have examined the differences in children’s play activities and with whom children play during recess across different levels of motor performance [21,22]. One study investigated the relationship between several play variables and fundamental motor skills in 3- to 5-year-old children and found that playing for relatively more time without equipment was associated with better locomotor and total motor skills (including both object control and locomotor skills), while spending relatively more time in locomotion activities was associated with lower locomotor and total motor skills [21]. Another study compared the playground activity of 6- to 10-year-old children with and without Developmental Coordination Disorder (DCD [22]), which is a neurodevelopmental disorder characterized by motor skill impairments [23]. It was found that children with DCD spent more time alone or in smaller groups, were onlookers more often, and were more involved in moving around the playground without participating in any game or activity compared to children without DCD. In conclusion, previous studies have investigated the relationship between play during recess and motor performance in 3- to 5-year old children [21] and in older atypically developing children [22], but this relationship has not yet been investigated in 6- to 8-year-old typically developing (TD) children. This is striking because it was found that during the ages of 6 to 8 years, children still mainly interact through motor activity during school recess, but this decreases and shifts towards conversation as they grow older [24]. Thus, the age period of 6 to 8 years may be regarded as a critical time for children to interact and play with peers, during which they can observe, imitate, practise, and improve their motor performance. Therefore, the aim of this study is to examine whether children’s play activities and with whom 6- to 8-year-old children play during recess are related to their motor performance.

However, a broad range of factors can be studied with regard to children’s play activities and with whom children play, such as the type of activity that is performed while playing as this determines the type of motor performance that can be practised [10,25]. Previous studies have found that children who participated more than their peers in locomotor and throwing activities, commonly classified as active behaviour, exhibited better gross motor performance [25,26,27]. Conversely, those who participated more in crafts and playing with small toys, often classified as sedentary behaviour, demonstrated better fine motor performance [10,28]. Therefore, the two activity-related aspects that will be focused on in this study are sedentary or active behaviour and activity type (e.g., physical play, fine motor play, etc.). It is expected that children who show more active behaviour and participate more in physical play such as games and sports will score better on gross motor performance and that children who show more sedentary behaviour and participate more in fine motor play such as sidewalk chalking and building a tower will score better on fine motor performance. Other factors regarding play that should be taken into account are the social aspects of play, as these determine with whom and in what manner children can practise and enhance their motor performance. One such factor is group size, because according to the social learning theory, behaviour is shaped by observing and imitating the behaviour of others [17], and the size of the group in which children play determines the number of children available to observe, imitate, and learn from. Playing in smaller groups may offer fewer opportunities for observation, imitation, and learning compared to larger groups, and vice versa. Another factor is the type of interactions children have. Imitation does not only serve the purpose of learning a new skill, but its goal can also be to affiliate with others and befriend them [29]. This is confirmed by the social learning theory that states that one of the motivators to imitate the behaviour of someone else is being able to identify with the other (i.e., feeling similar to the other, aspiring to be like the other, or liking the other) [17]. For this reason, the social interactions between children should be taken into account as children are more likely to imitate the behaviour of someone they perceive as warm and friendly than of someone who is perceived as unfriendly or unapproachable [29]. Therefore, the two social aspects that will be focused on in this study are group size and type of interaction, and it is expected that children playing in larger groups and having more positive social interactions will show better motor performance. Knowledge regarding the relationship between play during recess and motor performance, especially about how factors such as the type of activity performed and what type of interaction takes place are related to motor performance, may help teachers and policy makers to create stimulating situations during recesses in which motor performance can be practised and improved.

## 2. Materials and Methods

### 2.1. Study Sample

Data from the current study were collected as part of the ‘Uniek in je Motoriek’ [Being unique with regard to your motor skills] project. This cross-sectional project took place at Dutch primary schools in the northern regions of the Netherlands during regular school hours. Recruitment occurred via the participating schools. All children in the participating classes were invited, but only those that were able to join the regular physical education classes and whose parents had given written informed consent were included. Both parents and children were informed that they could opt out of the study at any given moment without having to explain their reasons. This project received approval from the Ethics Review Committee of the Department for Human Movement Sciences, University Medical Centre Groningen (research registry number 202000791).

### 2.2. Instruments

#### 2.2.1. Play Variables

Play on the playground during recess was observed using a modified version of the System for Observing Children’s Activity and Relationships during Play (SOCARP; [30]). The play variables that were observed during recess were sedentary or active behaviour, group size, activity type, and type of interaction (see Table 1 for all possible categories per play variable and their definitions; see Appendix A for the differences between the original and modified version).

Each participant was observed twice, on separate days, for 10 consecutive minutes during recess. The observations occurred in intervals of 10 s, alternating between 10 s of observation and 10 s of recording the observations. Every 10 s an audio signal was used to cue the transition between observation and recording. All observations were performed by extensively trained testers who were instructed to discretely position themselves on the playground and to perform the observation without the child noticing.

Sedentary or active behaviour, group size, and activity type were only observed and scored at the sound of the audio signal (i.e., momentary time sampling technique), while all interactions that the participant was engaged in during the entire 10 s of observation were recorded (i.e., partial interval recording technique). Not every variable had mutually exclusive categories, and thus, some of the categories had to be given priority over other categories in order to score only one category per observation interval. For example, activity type was only scored when the recording cue sounded, but still multiple activities were possible at once, such as having a conversation while walking. Therefore, onlooking was given priority over all other activities, although it was taken into account whether the child was onlooking while being involved in another activity or onlooking without being involved in another activity. Physical, imaginative, and fine motor play were given priority over verbal play (e.g., talking while playing a soccer game was scored as physical play), while all activity types were given priority over non-play activities (e.g., having a conversation while walking was scored as verbal play). Another variable that was not mutually exclusive was the type of interaction as all interactions that the participant engaged in throughout the observation period were recorded. To make this variable mutually exclusive, anti-social interaction was given priority over the other types of interaction, and it was only possible to score no interaction when neither anti-social nor social interactions were observed during that interval.

A previous study investigating the original tool has shown an acceptable inter-rater reliability, with agreement percentages between 88% and 90% for all play variables, and has proven the construct validity of the tool through a significant correlation with accelerometer data [30].

#### 2.2.2. Motor Performance

Motor performance was measured with age band 1 (i.e., for the 6-year-olds) and age band 2 (i.e., for the 7- and 8-year-olds) from the Dutch version of the Movement Assessment Battery for Children 2 (MABC-2) [33]. This test battery assesses three components of motor performance: Manual Dexterity (three items), Aiming and Catching (two items), and Balance (three items). For age band 1, the Manual Dexterity items were ‘Posting Coins’, ‘Threading Beads’, and ‘Drawing Trail’; Aiming and Catching included ‘Catching a Bean Bag’ and ‘Throwing a Bean Bag’; and Balance involved ‘One-Leg Stand’, ‘Walking on Toes’, and ‘Jumping on Mats’. For age band 2, Manual Dexterity included ‘Place Pegs’, ‘Threading Lace’, and ‘Drawing Trail’; Aiming and Catching remained the same; and Balance involved ‘One-Board Balance’, ‘Walking Heel to Toe Forward’, and ‘Hopping on Mats’.

The raw scores for each component were corrected for age and converted into standard scores according to the norms as described in the Dutch manual of the MABC-2 [33]. The MABC-2 provides a traffic-light system that can be used to classify children based on their standard scores according to the following categories: (1) the red zone (i.e., percentile score ≤ 5, indicating significant motor difficulties), (2) the orange zone (i.e., percentile score between >5 and ≤16, indicating risk of developing motor difficulties), and (3) the green zone (i.e., percentile score > 16, indicating no motor difficulties). In the current study, children were divided into two groups for each motor performance component, with those scoring in the orange and red zone in one group and children scoring in the green zone in the other group.

Age band 1 and 2 of the MABC-2 have shown good-to-excellent reliability (i.e., intra-rater and inter-rater reliability) and excellent validity (i.e., content, structural, and criterion validity) [34,35]. Moreover, the Dutch version of age band 1 has acceptable-to-good internal consistency [36] and the Dutch version of age band 2 has a moderate concurrent validity with the Bruininks–Oseretsky Test of Motor Proficiency [37].

#### 2.2.3. Confounding Variables

The potential confounding variables that were taken into account were age, sex, and weekly physical activity (PA). The age and sex of the children were registered by the parents while filling out the written informed consent. Weekly PA was measured using accelerometers (ActiGraphs GT9X Link, Pensacola, FL, USA). These were worn on the right hip during all waking hours for an entire week, except while bathing or swimming. The data were analysed from 15 s epoch recordings using ActiLife software (v.6.13.4). Periods of at least 60 min with zero counts were defined as non-wearing time. Days with less than 480 min of wear time were not taken into account, and children with fewer than three valid wear days were excluded from the analysis. Weekly behaviour was divided into either sedentary behaviour or light, moderate, and vigorous PA based on the cut-off points suggested by Evenson and colleagues [38].

### 2.3. Statistical Analysis

Before describing the analyses, it is important to elaborate on the nature of the data. Sex and the MABC-2 scores are treated as binomial scores (i.e., either no motor difficulties or being at risk of/having significant motor difficulties), while weekly PA and the play variables are considered compositional data. Compositional data convey relative information, because an increase in the time spent in one category automatically means a decrease in time spent in other categories. Therefore, these data are only meaningful when interpreted relative to each other (i.e., compositional data analysis), and thus, traditional multivariate statistics cannot be applied directly to these data [39,40,41].

All analyses were performed using R v4.3.1 [42] and the significance level for all analyses was set at 0.05. To deal with the compositional nature of some of the variables and to be able to make use of the traditional multivariate statistics, the data needed to be transformed. First, all zeros in the dataset were replaced using the Bayesian-Multiplicative replacement method from the ‘zCompositions’ package v1.5.0-4 [43]. This was only performed directly when the proportion of zeros in each category was below the threshold of 80%. If this was not the case, categories had to be merged first to decrease the proportion of zeros. Then, the variables were assigned as compositional data, after which an isometric log-ratio (ilr) transformation was performed. This created pivot coordinates, which are coordinates expressing the relative information of the compositional data. The first coordinate captures the contributions of the first category of each variable relative to the remaining categories of that variable and is thus easily interpreted, while the consecutive coordinates treat the redundant information in a controlled but less interpretable manner [41]. Therefore, different sets of pivot coordinates were created so that there was a first pivot coordinate for each category (i.e., interpretable information about that category relative to the other categories) which was used in further analyses. Both the classification as compositional data and the transformation were carried out using the ‘Compositions v2.0-6’ package [44].

Subsequently, the descriptive statistics were calculated, including the arithmetic means and the standard deviations for all variables and the geometric means and pairwise variation matrices for the compositional play variables. The geometric mean minimizes the relative distance between datapoints instead of the absolute distance, and the pairwise variation is investigated, because as one part varies in compositional data, at least one other part must also vary [41].

To investigate the relationships between the play variables and motor performance, bivariate correlations and logistic regression analyses were performed. Prior to the analyses, the assumptions of linearity between the continuous independent variables and the log odds, the absence of multicollinearity (i.e., variance inflation factor below 5 [45]), and the absence of outliers were checked and met. First, the bivariate correlations were calculated with Pearson correlations when both variables were continuous, with point biserial correlations when one variable was dichotomous, and with Phi correlations when both variables were dichotomous [46]. A correlation coefficient was considered weak between 0.1 and 0.3, moderate between 0.3 and 0.6, and strong between 0.6 and 1.0. Subsequently, three logistic regressions using the Enter method were performed, with each motor performance component as a dependent variable, the first pivot coordinates of each category of the play variables as independent variables, and age, sex, and weekly PA as potential confounders using the ‘stats v4.3.1’ package.

## 3. Results

The study sample consisted of 86 participants (mean age 6.92 ± 0.62 years, 58.1% boys) from five primary schools, after 4 participants (mean age = 7.33 ± 0.58 years, 25.0% boys) were removed because they either had missing data on the MABC-2 (*n* = 2) or were observed for less than 50% of the time during one of the two or both observation moments (*n* = 2). When applying the zero replacement method, it was discovered that the proportion of zeros in the activity type category ‘fine motor play’ exceeded the threshold of 80%. Therefore, it was decided to merge this category with the ‘imaginative play’ category, as fine motor play mostly entailed constructing something in the sandpit or with small wooden chips, which, just like ‘imaginative play’, required imagination and creativity [47].

The descriptives, including the arithmetic mean and standard deviation for all study variables and the geometrical mean for the compositional variables, are shown in Table 2. When looking at these descriptives, it can be seen that according to the norm scores (i.e., the lowest 16% of the scores are classified as the orange and red zone [33]), many children have scores in the red or orange zone for the Aiming and Catching component, while a normal distribution of scores is found for the Manual Dexterity and Balance components. The pairwise variation matrices for the compositional variables can be found in Appendix B.

The results of the bivariate correlations (Table 3) showed that age was not significantly related to either the play variables or the motor components, while weekly PA and sex were significantly related to several of these variables. Therefore, it was decided to only include weekly PA and sex as potential confounders in the subsequent analyses. None of the play variables correlated significantly with any of the motor components.

The results of the logistic regressions (Table 4) showed that sedentary or active behaviour significantly predicted performance in the Aiming and Catching component. Specifically, showing more sedentary behaviour (and consequently less active behaviour) was associated with higher odds of being in the green zone (i.e., indicating no motor difficulties) for Aiming and Catching. Furthermore, none of the other play variables were significant predictors for any of the motor components.

## 4. Discussion

The aim of this study was to examine whether children’s play activities and with whom 6- to 8-year-old children play during recess are related to their motor performance. It was hypothesized that children who show more active behaviour and participate more in physical play such as games and sports will show better gross motor performance and that children who show more sedentary behaviour and participate more in fine motor play such as sidewalk chalking and building a tower will score better on fine motor performance. Furthermore, it was expected that children who played in larger groups and had more positive social interactions would score better on both gross and fine motor performance. However, contrary to our hypotheses, it was found that spending more time in sedentary behaviour rather than in active behaviour was related to higher odds of being in the green zone (i.e., no motor difficulties) for the Aiming and Catching component (i.e., a gross motor skill) of the MABC-2. The play variable sedentary or active behaviour was not related to the other motor components. Furthermore, the play variables group size, activity type, and type of interaction during play were not related to any of the motor components.

A key finding of this study was that children who spent more time in sedentary behaviour during recess rather than in active behaviour were less likely to have motor difficulties in the Aiming and Catching component of the MABC-2. Previous studies investigating the relationship between sedentary or active behaviour and motor performance in 6- to 8-year-olds found mixed results [26,48,49]. Two studies found more moderate-to-vigorous physical activity (MVPA) throughout the day to be related to better object control skills [26,48]. However, another study found more MVPA throughout the day to be related to worse Aiming and Catching skills [49], which is in concurrence with the findings of the current study. It is important to note that these previous studies looked at MVPA throughout the day [26,48,49], whereas in the current study, active behaviour was assessed during recess and encompassed both light PA and MVPA. It was decided to take both light PA and MVPA into account because children spent a relatively large portion of their daily PA in light PA [50,51], and thus, light PA might play a big role in how the play variable sedentary or active behaviour is related to motor performance. The contrasting findings seem to be linked to the methods of assessing ball skills. The studies that have found more MVPA to be related to better ball skills used the object control items of the Test for Gross Motor Development-3 (TGMD-3 [26,48]), while the studies that found more MVPA to be related to worse ball skills [49], including the current study, used the Aiming and Catching component of the MABC-2. One difference between these tests is that the TGDM-3 is a process-oriented test that focuses on how a movement is executed and describes the qualitative movement patterns, while the MABC-2 is a product-oriented test that evaluates the outcome of a movement (i.e., time to perform a task or correct executions) [52,53]. However, it is unclear if and how this explains the different findings. Another difference between the tests is the type of ball skills that are assessed. The TGMD-3 focuses on fundamental movement skills that are needed to participate in sports [54] and thus includes both static and more dynamic ball skills (i.e., one- and two-handed striking, dribbling, overhand throwing, underhand throwing, catching, and kicking [55]), whereas the MABC-2 contains one static aiming task and one static catching task [33]. Therefore, children who show relatively more active behaviour might score better on a test that measures more dynamic ball skills, while children who show relatively more sedentary behaviour might score better on a test that measures only static ball skills. Furthermore, an additional explanation for this relationship might be found in assuming that children who show relatively more sedentary behaviour during recess are likely to continue such habits during leisure time. Videogaming, which is considered to be a sedentary activity [56], has become a highly prevalent leisure activity, with 87% of Dutch primary school children reporting playing videogames occasionally and 33% of children reporting playing almost daily [57]. In turn, playing videogames has been shown to improve visuomotor performance, including reaction time, control precision, and response amplitude [58,59,60], which are essential skills for aiming and catching [61]. Consequently, gaming could provide an alternative pathway through which sedentary behaviour could lead to better aiming and catching skills.

The play variable activity type was not related to any of the motor components in the current study, which is contradictory to previous research. One study investigated the relationship between activity type (i.e., categorized as active games with equipment, active games without equipment, sedentary play, quiet play, and locomotion) and fundamental motor skills in 3- to 5-year-old children and found that spending more time in active games without equipment was associated with better locomotor skills, while spending more time in locomotion was associated with worse locomotor skills [21]. Another study investigated the relationship between activity type and motor performance in the opposite direction than the current study [22]. The mentioned study compared activity type (i.e., categorized as formal team games, informal team games, fine motor rule games, gross motor games with evolving rules, skill mastery, rough and tumble play, negative social interaction, stationary fantasy play, moving fantasy play, being stationary with no context, and moving with no context) between 6- to 10-year-old TD children and children with DCD. It was found that TD boys played more formal team games than boys with DCD, TD girls played more informal team games than girls with DCD, and boys and girls with DCD were onlookers more often compared to TD children [22]. The findings from these previous studies seem to suggest that activity type is related to motor performance, although this was not confirmed in the current study. One of the differences between the current study and these other studies is that one other study investigated the differences for boys and girls separately [22]. This may be important to consider as boys and girls are often involved in different activities [62]. Boys tend to play more roughly and physically, while girls tend to be more involved in play that requires verbal interaction. This is confirmed in the current study as it was found that being a boy was moderately correlated to spending more time in physical play alone (r = 0.37) and weakly correlated to spending less time in imaginative/fine motor play (r = −0.30). Thus, playing with the same or opposite sex might provide different experiences and opportunities to practise motor performance [22,63]. Therefore, group composition (i.e., whether children play with other children of the same or opposite sex) should be taken into consideration in future research.

The play variable group size was also not related to any of the motor components. This is in concurrence with a previous study that found no relationship between group size and object control or locomotor skills in 3- to 5-year-old TD children [21]. One explanation might be that this play variable in itself is not related to motor performance, but possibly is related if the other play variables are taken into account as well. The isolated variable group size only described whether a child played in a small group or a large group, but did not clarify if the children were actually playing and interacting with each other or which activity was performed and thus which type of motor performance could be practised [10,25]. Consequently, it is recommended for future research to investigate whether the combinations of play variables (i.e., interactions between the play variables) are related to motor performance, so that the variables are investigated as an integrated whole rather than in isolation. Another explanation could be that additional play variables should be taken into account. In the current study, it was found that spending more time in a large group (and thus spending less time alone or in small groups) was moderately correlated to spending more time onlooking while being involved in an activity (*r* = 0.33). In scenarios such as when children are playing football in a large group, not every child can chase, kick, or steal the ball at the same time, and so there are also children in the group watching others play rather than actively playing and practising their motor performance themselves. Therefore, whether a child is actively participating in the activity they are doing or not should be taken into account. Another study found a relationship between motor performance and group size [22]. However, that study investigated how poor motor performance affected the size of the group in which children played, which is the opposite direction to the relationship that was investigated in the current study. This other study compared group size between 6- to 10-year-old TD children and children with DCD and found that children with DCD spend more time alone, more time playing with only one other child, and less time playing in groups with eight or more compared to TD children [22]. The different findings might suggest that the relationship between group size and motor performance is more pronounced in children with DCD, while TD children do not seem to be limited to participate in groups. Another explanation could be that the previous study was performed more than twenty years before the current study [22]. Inclusivity in education has been improving rapidly during the past few decades [64], and thus, it could be that in the meantime, inclusivity in schools and on the playground has improved and that motor skills are no longer a barrier to TD children being included in play.

The absence of associations between the type of interaction children had and any of the motor components might be due to the controlled environment during recess. The type of interaction was hypothesized to be related to motor performance because according to the social learning theory, the chances of observational learning increase when the person to learn from is perceived as warm and friendly [17]. Therefore, it was expected that more social interaction would be related to better motor performance. However, this relationship was not confirmed in our study, possibly due to the predominance of social interactions (geometrical mean = 82.70% of the time), while almost no anti-social interactions were observed (geometrical mean = 0.69% of the time). The prevalence of mainly social interactions could be attributed to familiarity among children, as a previous study showed that children who played with familiar peers tended to have higher levels of social interactions and cooperative play [65]. The absence of anti-social interactions could be explained by the presence of teachers during every observed recess who could intervene and interrupt any potential anti-social interactions. This suggests that the limited diversity in the types of interactions could be the reason why the possible relationship between type of interaction and motor performance is not evident in the relatively controlled (i.e., supervised) situation during recess. It might be that the type of interactions children engage in could be related to motor performance in a different setting, for instance on a playground outside of school where children have the opportunity to play completely freely and do not always know each other.

### Strengths and Limitations

The most important strength of this study was the comprehensiveness of the measurements, which encompassed observations on the playground on a broad range of play variables and across multiple days. This allowed us to capture many different aspects of play at once. Furthermore, the observations were performed during recess as this is an important moment during the school day for children to play and interact with peers [20] and a potentially important time to practise and improve their motor performance skills. Another strength of this study was the use of compositional data analysis, which took the relative nature of the data into account (i.e., the data are only meaningful when interpreted relative to each other because an increase in the time spent in one category automatically means a decrease in time spent in other categories). However, this study also has some limitations that need to be considered. Observed behaviours are very likely to be impacted by contextual factors that could vary from day to day, such as weather conditions or the presence of favourite peers [66]. Therefore, to obtain an accurate overview of how children generally play on the playground, they should be observed multiple times under different conditions. In the current study, efforts were made to minimize this risk by observing each participant twice, on separate days, for 10 consecutive minutes. However, future studies should consider even further increasing the number of observation sessions and extending the duration to capture a broader range of behaviours and account for day-to-day variations. Another important factor to consider is the potential sex difference in the relationship between play during recess and motor performance. The correlation table in the current study suggests that differences between boys and girls exist in both motor performance and the play variables. Although sex was included as a confounder in the analyses, this approach does not fully capture how the relationship differs between sexes. Future studies should include a larger sample size to allow for separate analyses for boys and girls. Finally, the relationship between play during recess and motor performance was investigated unidirectionally, even though it can be hypothesized that this relationship is bidirectional. The activity performed determines the type of motor performance that can be practised; however, the type of motor performance that children excel in might also determine which activity they choose to participate in [10,25]. Similarly, children may improve their motor performance through interaction with peers, but the level of motor performance might also influence if and how children interact with peers [22,67]. Therefore, future research should take the bidirectional nature of this relationship into account.

## 5. Conclusions

Contrary to our hypotheses, children who showed more sedentary behaviour rather than active behaviour were less likely to have motor difficulties in the Aiming and Catching component of the MABC-2. This could be explained by the static nature of the assessment used in the current study. However, another explanation might be that gaming, which is a prevalent sedentary activity outside of school, might improve visuomotor performance, which in turn might improve aiming and catching skills. Also contrary to our hypotheses, none of the other play variables were related to motor performance. This might suggest that inclusivity in schools and on the playground has improved so much that motor skills are no longer a barrier to TD children being included in play. It remains to be investigated whether interactions between the play variables are related to motor performance and if additional play variables, such as group composition and active participation, are related to motor performance. Furthermore, future research should try to enhance the generalizability of the findings by increasing the duration and frequency of the observations and should take the bidirectional nature of the relationship into account.

## Figures and Tables

**Table 1:** All play variables, their categories, and the definition of each category of the modified version of the System for Observing Children’s Activity and Relationships during Play [30].

Play Variables	Categories	Definition of Each Category
Sedentary or active behaviour	Sedentary	Included lying, sitting, and standing
	Active	Included walking and moderate-to-vigorous physical activity
Group size	Alone	The observed child did not play with other children
	Small	Group included 2 to 4 children including the observed child
	Large	Group included 5 or more children including the observed child
Activity type ^a^	Physical play with others	All physical play activities (e.g., sports, games, functional play, and rough and tumble play) performed in a group
	Physical play alone	Physical play as defined above but performed alone
	Verbal play	All games and non-games involving talking, such as having a conversation, singing, or deciding the rules and roles for a game
	Imaginative play	Role enactment or acting games
	Non-play activities	Activities without a clear play aspect, such as walking around, eating, or tying shoelaces
	Fine motor play	All play that requires the child to use the smaller muscle groups and to show a high degree of precision
	Onlooking—being involved	Watching the play of others while being involved in the same or another activity (e.g., watching someone else kick the ball while being involved in the same soccer game)
	Onlooking—not being involved	Watching the play of others while not being involved in the same or another activity (e.g., standing on the side watching others play a soccer game)
Type of interaction	Social interaction	Includes both physical and non-physical pro-social (i.e., positive interactions such as providing encouragement or holding hands) and social interactions (i.e., neutral interactions during which children play nicely together without any positive or negative interactions)
	Anti-social interaction	Both physical and non-physical negative interactions such as taking equipment away or name calling
	No interaction	The child does not interact with anyone

Note. ^a^ Activity type was scored according to a classification developed by Bishop and Curtis [31], which was slightly modified based on other research [22,32].

**Table 2:** The descriptives including the arithmetic mean, standard deviation, percentages, and geometric means of the study variables.

Variables	Percentage	Mean	SD	Geometric Mean
Confounding variables				
Age (years)		6.92	0.62	
Sex (% boys)	58.14			
Weekly PA				
Sedentary (% of time)		54.26	6.00	54.30
Light, moderate, and vigorous (% of time)		45.74	6.00	45.70
Manual Dexterity—MABC-2				
Orange and red zone	17.44			
Green zone	82.56			
Aiming and Catching—MABC-2				
Orange and red zone	29.41			
Green zone	70.59			
Balance—MABC-2				
Orange and red zone	17.44			
Green zone	82.56			
Sedentary or active behaviour				
Sedentary behaviour (% of time)		51.69	19.10	51.75
Active behaviour (% of time)		48.31	19.10	48.25
Group size				
Alone		16.84	16.53	14.74
Small group (% of time)		55.59	25.89	64.93
Large group (% of time)		27.57	25.21	20.33
Activity type				
Physical play with others (% of time)		26.16	24.48	24.09
Physical play alone (% of time)		1.87	3.19	1.08
Verbal play (% of time)		20.54	16.53	21.71
Imaginative/fine motor play (% of time)		7.84	12.01	4.54
Non-play activities (% of time)		15.44	10.42	19.09
Onlooking—being involved (% of time)		6.78	10.51	4.86
Onlooking—not being involved (% of time)		21.36	17.11	24.64
Type of interaction				
None (% of time)		21.43	18.29	16.61
Social (% of time)		76.29	18.32	82.70
Anti-social (% of time)		2.32	3.84	0.69

Note. MABC-2 = Movement Assessment Battery for Children-2. The proportion of zeros in the ‘fine motor play’ category exceeded the threshold of 80% and this category was therefore merged with the ‘imaginative play’ category.

**Table 3:** The bivariate correlation matrix for the key and potential confounding variables.

Variables	2	3	4	5	6	7	8	9	10	11	12	13	14	15	16	17	18	19	20	21	22
1. Age (months)	0.14	0.02	−0.02	0.01	0.12	0.01	0.02	−0.02	0.01	0.07	−0.07	−0.17	0.03	−0.00	0.03	0.16	−0.05	−0.01	−0.04	0.03	−0.00
2. Sex		−0.25 *	0.25 *	0.27 *	−0.02	0.27 *	−0.31 **	0.31 **	0.52 ***	−0.47 ***	0.08	−0.06	0.37 ***	0.12	−0.30 **	−0.14	0.10	−0.16	0.26 *	−0.41 ***	0.21
3. Weekly PA—sedentary (ilr)			−10.00 ***	−0.29 **	−0.01	−0.04	−0.07	0.29 **	−0.29 **	0.09	0.19	−0.25 *	−0.2	−0.03	−0.03	0.03	0.12	−0.23 *	0.32 **	0.27 *	0.33 **
4. Weekly PA—light, moderate, and vigorous (ilr)				0.29 **	0.01	0.04	0.07	−0.29 **	0.29 **	−0.09	−0.19	0.25 *	0.2	0.03	0.03	−0.03	−0.12	0.23 *	−0.32 **	−0.27 *	−0.33 **
5. Manual Dexterity MABC-2					0.11	0.43 ***	−0.20	0.20	0.03	−0.10	0.08	0.08	−0.03	0.11	−0.18	0.12	0.01	−0.12	−0.06	−0.17	0.20
6. Aiming and Catching MABC-2						−0.03	0.14	−0.14	−0.03	0.16	−0.13	0.02	−0.10	0.06	−0.07	0.14	−0.06	0.01	0.04	−0.13	0.10
7. Balance MABC-2							−0.19	0.19	0.18	−0.19	0.05	0.06	0.12	−0.01	−0.02	−0.06	−0.05	−0.07	−0.01	−0.20	0.19
Sedentary or active behaviour																					
8. Sedentary behaviour (ilr)								−10.00 ***	−0.13	0.38 ***	−0.27 *	−0.29 **	−0.35 ***	−0.04	0.30 **	−0.04	−0.14	0.62 ***	0.13	0.21	−0.28 **
9. Active behaviour (ilr)									0.13	−0.38 ***	0.27 *	0.29 **	0.35 ***	0.04	−0.30 **	0.04	0.14	−0.62 ***	−0.13	−0.21	0.28 **
Group size																					
10. Alone (ilr)										−0.33 **	−0.40 ***	−0.41 ***	0.51 ***	0.14	−0.39 ***	−0.09	−0.07	0.17	0.71 ***	−0.30 **	−0.19
11. Small group (ilr)											−0.73 ***	−0.24 *	−0.28 **	−0.29 **	0.37 ***	0.33 **	−0.29 **	0.43 ***	0.05	0.22 *	−0.24 *
12. Large group (ilr)												0.53 ***	−0.10	0.18	−0.08	−0.26 *	0.33 **	−0.54 ***	−0.57 ***	0.01	0.38 ***
Activity type																					
13. Physical play with others (ilr)													−0.51 ***	0.09	−0.06	0.11	−0.04	−0.51 ***	−0.40 ***	−0.05	0.32 **
14. Physical play alone (ilr)														−0.24 *	−0.07	−0.39 ***	0.25 *	−0.19	0.35 ***	−0.20	−0.05
15. Verbal play (ilr)															−0.80 ***	0.26 *	−0.17	−0.19	0.02	−0.01	−0.00
16. Imaginative/fine motor play (ilr)																−0.36 ***	0.18	0.22 *	−0.24 *	0.18	−0.01
17. Non-play activities (ilr)																	−0.72 ***	0.05	0.02	0.09	−0.10
18. Onlooking—being involved (ilr)																		−0.40 ***	−0.19	−0.02	0.15
19. Onlooking—not being involved (ilr)																			0.32 **	0.05	−0.27 *
Type of interaction																					
20. None (ilr)																				−0.25 *	−0.44 ***
21. Social (ilr)																					−0.76 ***
22. Anti-social (ilr)																					

Note. Sex: 0 = girls; 1 = boys; MABC-2 = Movement Assessment Battery for Children-2; ilr = pivot coordinate (i.e., one part of compositional data), which should be interpreted relative to the other categories. Bivariate correlations were calculated with Pearson correlations when both variables were continuous, with point biserial correlations when one variable was dichotomous, and with Phi correlations when both variables were dichotomous [46]. A correlation coefficient is considered weak between 0.1 and 0.3, moderate between 0.3 and 0.6, and strong between 0.6 and 1.0. * *p* < 0.05. ** *p* < 0.01. *** *p* < 0.001.

**Table 4:** The logistic regression analyses predicting the MABC-2 component scores from the play variables.

Variables	Manual Dexterity		Aiming and Catching		Balance	
	OR	95% CI	OR	95% CI	OR	95% CI
Intercept	4.37	[0.29, 79.80]	2.51	[0.30, 22.69]	3.00	[0.23, 43.73]
Sex	8.48 *	[1.34, 86.55]	1.19	[0.31, 4.70]	4.47	[0.73, 42.66]
Weekly PA						
Sedentary (ilr)	0.03	[0.00, 3.18]	1.18	[0.03, 56.09]	13.50	[0.18, 1770.38]
Light, moderate, and vigorous (ilr)	28.95	[0.31, 4774.14]	0.85	[0.02, 36.09]	0.07	[0.00, 5.47]
Sedentary or active behaviour						
Sedentary behaviour (ilr)	0.53	[0.08, 2.84]	3.72 *	[1.04, 14.85]	0.43	[0.06, 2.31]
Active behaviour (ilr)	1.90	[0.35, 12.04]	0.27 *	[0.07, 0.96]	2.34	[0.43, 16.40]
* Group size*						
Alone (ilr)	0.67	[0.14, 2.80]	0.89	[0.32, 2.50]	2.52	[0.68, 12.16]
Small group (ilr)	1.02	[0.39, 2.75]	1.62	[0.81, 3.40]	0.68	[0.26, 1.57]
Large group (ilr)	1.48	[0.49, 4.85]	0.69	[0.33, 1.41]	0.58	[0.19, 1.63]
Activity type						
Physical play with others (ilr)	0.82	[0.38, 1.75]	1.33	[0.76, 2.42]	1.04	[0.49, 2.29]
Physical play alone (ilr)	0.94	[0.41, 2.17]	1.07	[0.61, 1.89]	0.84	[0.38, 1.72]
Verbal play (ilr)	1.14	[0.56, 2.57]	1.06	[0.65, 1.74]	1.07	[0.54, 2.32]
Imaginative/fine motor play (ilr)	0.44	[0.12, 1.19]	0.59	[0.27, 1.18]	1.56	[0.69, 3.78]
Non-play activities (ilr)	2.22	[0.83, 7.53]	1.41	[0.80, 2.67]	0.82	[0.38, 1.84]
Onlooking—being involved (ilr)	0.96	[0.45, 1.94]	1.24	[0.71, 2.22]	0.60	[0.26, 1.24]
Onlooking—not being involved (ilr)	1.20	[0.51, 3.00]	0.64	[0.33, 1.19]	1.38	[0.57, 3.62]
Type of interaction						
None (ilr)	1.11	[0.32, 3.73]	0.91	[0.35, 2.31]	0.44	[0.10, 1.59]
Social (ilr)	0.86	[0.39, 1.86]	0.79	[0.43, 1.42]	1.14	[0.51, 2.75]
Anti-social (ilr)	1.06	[0.52, 2.25]	1.39	[0.79, 2.56]	1.96	[0.93, 4.71]
Total model						
χ^2^ (df)	18.90 (13)		11.42 (13)		15.78 (13)	
*p*-value	0.126		0.576		0.261	
Fit	87.21%		74.12%		81.40%	
Sensitivity	100.00%		91.67%		95.77%	
Specificity	26.67%		32.00%		13.33%	

Note. OR = Odds Ratio; CI = confidence interval; Sex: 0 = girls and 1 = boys; ilr = pivot coordinate (i.e., one part of compositional data), which should be interpreted relative to the other categories. The OR serves both as a transformed version of the coefficient (i.e., log odds) and as the unstandardized effect size of the logistic regression. * *p* < 0.05.

## Data Availability

The data presented in this study are available on request from the corresponding author. The data are not publicly available because parental consent was only given for the use of such data by the researchers directly involved in the ‘Uniek in je Motoriek’ [Being unique with regard to your motor skills] project.

## Appendix A. Modifications to SOCARP

**Table A1:** The modified version of the System for Observing Children’s Activity and Relationships during Play (SOCARP).

Play Variables	Changes	Categories in the Original Version	Categories in the Modified Version
Sedentary or active behaviour	Some of the categories of this play variable were merged	Lying Sitting Standing Walking/moderate Very active/vigorous	Sedentary (i.e., lying, sitting, and standing) Active (i.e., walking/moderate activity and vigorous activity)
Group size	Some of the categories of this play variable were merged	Alone Small (i.e., 2 to 4 people) Medium (i.e., 5 to 9 people) Large (i.e., 10 or more people)	Alone Small (i.e., 2 to 4 children) Large (i.e., 5 or more children)
Activity type	The categories of this play variables were completely modified	Sports Active games Locomotion Sedentary	Physical play with others Physical play alone Verbal play Imaginative play Non-play activities Fine motor play Onlooking—being involved Onlooking—not being involved
Type of interaction	Some of the categories of this play variable were merged	Pro-social physical Pro-social non-physical Anti-social physical Anti-social non-physical None Ignore	Social interaction (i.e., both physical and non-physical) Anti-social interaction (i.e., both physical and non-physical) No interaction

## Appendix B. Pairwise Variation Matrices for the Compositional Variables

**Table A2:** The pairwise variation matrix for the following confounding variable: weekly physical activity (PA).

	Sedentary	Light, Moderate, and Vigorous
Sedentary	0.00	0.06
Light, moderate, and vigorous	0.06	0.00

**Table A3:** The pairwise variation matrix for the following play variable: sedentary or active behaviour.

	Sedentary	Active
Sedentary	0.00	0.79
Active	0.79	0.00

**Table A4:** The pairwise variation matrix for the following play variable: group size.

	Alone	Small	Large
Alone	0.00	1.82	3.45
Small	1.82	0.00	3.26
Large	3.45	3.26	0.00

**Table A5:** The pairwise variation matrix for the following play variable: activity type.

	Physical Play with Others	Physical Play Alone	Verbal Play	Imaginative and Fine Motor Play	Non-Play Activities	Onlooking—Being Involved	Onlooking—Not Being Involved
Physical play with others	0.00	3.24	3.87	5.90	3.02	3.56	3.37
Physical play alone	3.24	0.00	4.48	5.93	3.41	3.23	3.25
Verbal play	3.87	4.48	0.00	3.27	1.22	4.06	2.38
Imaginative and fine motor play	5.90	5.93	3.27	0.00	2.71	3.86	3.17
Non-play activities	3.02	3.41	1.22	2.71	0.00	3.28	1.66
Onlooking—being involved	3.56	3.23	4.06	3.86	3.28	0.00	2.52
Onlooking—not being involved	3.37	3.25	2.38	3.17	1.66	2.52	0.00

**Table A6:** The pairwise variation matrix for the following play variable: type of interaction.

	No Interaction	Social Interaction	Anti-Social Interaction
No interaction	0.00	1.74	3.83
Social interaction	1.74	0.00	3.27
Anti-social interaction	3.83	3.27	0.00
